# Transcription Is Required to Establish Maternal Imprinting at the Prader-Willi Syndrome and Angelman Syndrome Locus

**DOI:** 10.1371/journal.pgen.1002422

**Published:** 2011-12-29

**Authors:** Emily Y. Smith, Christopher R. Futtner, Stormy J. Chamberlain, Karen A. Johnstone, James L. Resnick

**Affiliations:** Department of Molecular Genetics and Microbiology, University of Florida College of Medicine, Gainesville, Florida, United States of America; University of Cambridge, United Kingdom

## Abstract

The Prader-Willi syndrome (PWS [MIM 17620]) and Angelman syndrome (AS [MIM 105830]) locus is controlled by a bipartite imprinting center (IC) consisting of the PWS-IC and the AS-IC. The most widely accepted model of IC function proposes that the PWS-IC activates gene expression from the paternal allele, while the AS-IC acts to epigenetically inactivate the PWS-IC on the maternal allele, thus silencing the paternally expressed genes. Gene order and imprinting patterns at the PWS/AS locus are well conserved from human to mouse; however, a murine AS-IC has yet to be identified. We investigated a potential regulatory role for transcription from the *Snrpn* alternative upstream exons in silencing the maternal allele using a murine transgene containing *Snrpn* and three upstream exons. This transgene displayed appropriate imprinted expression and epigenetic marks, demonstrating the presence of a functional AS-IC. Transcription of the upstream exons from the endogenous locus correlates with imprint establishment in oocytes, and this upstream exon expression pattern was conserved on the transgene. A transgene bearing targeted deletions of each of the three upstream exons exhibited loss of imprinting upon maternal transmission. These results support a model in which transcription from the *Snrpn* upstream exons directs the maternal imprint at the PWS-IC.

## Author Summary

Prader-Willi and Angelman syndromes are neurobehavioral disorders arising from dysregulation of a cluster of imprinted genes located at chromosome 15q11–q13. PWS results from the absence of paternally expressed genes and AS from the absence of maternally expressed genes. Two elements, termed the PWS-IC and the AS-IC, are responsible for allele-specific gene expression. The PWS-IC activates expression of paternally expressed genes, while the AS-IC is thought to silence the PWS-IC in the female germ line, rendering it inactive on the future maternal allele. Mouse models have been effective for studying the IC-directed regulation of this locus; however, the murine AS-IC has yet to be characterized. In this study, we have determined the identity of the AS-IC and investigated how it functions to inactivate the PWS-IC. Our results suggest that the murine AS-IC consists of several promoters that direct expression of transcripts through the PWS-IC in oocytes. Thus, faulty transcription in oocytes may lead to AS imprinting defects.

## Introduction

Genomic imprinting is an epigenetic phenomenon that occurs at a subset of chromosomal regions and results in parent-of-origin specific monoallelic gene expression. Imprinted genes are frequently found in clusters, coordinately regulated by imprinting centers (ICs) that direct allele-specific differences in transcription, DNA methylation, histone modifications and replication timing [1]–[4]. Appropriate control of imprinted gene expression is vital to growth and development and errors in imprinting may lead to developmental disorders or embryonic lethality.

Prader-Willi syndrome (PWS) and Angelman syndrome (AS) are distinct neurogenetic disorders resulting from improper gene expression from an imprinted domain on chromosome 15q11–q13, the PWS/AS locus. Mutations arising from the maternal chromosome that lead to a loss of UBE3A function are sufficient to cause AS [5], [6]. PWS results from the loss of multiple paternal gene products encoded at the PWS/AS locus. A bipartite IC, comprised of the PWS-IC and the AS-IC, regulates both epigenetic reprogramming and allele-specific gene expression at the PWS/AS locus [7], [8]. Prevailing models of IC function suggest that the PWS-IC is a positive element that activates gene expression from the paternal allele. The AS-IC acts as a negative element to direct inhibitory epigenetic modifications at the PWS-IC during oogenesis, thereby silencing the paternally expressed genes on the future maternal allele [8]–[10]. A subset of individuals with PWS or AS bear microdeletions that disrupt gene expression at the PWS/AS locus. The shortest regions of overlap for these microdeletions define the boundaries of the IC. The PWS-IC is located in a region of 4.3 kb, just 5′ to and including exon 1 of *SNRPN*, while the AS-IC is contained within 0.88 kb approximately 35 kb upstream of the PWS-IC [11], [12]. Notably, within the 0.88 kb of AS-IC sequence reside two of several alternative upstream exons of *SNRPN* [13], [14]. These upstream exons are postulated to play a role in silencing the PWS-IC on the maternal allele [8], [15].

**Figure 1:**
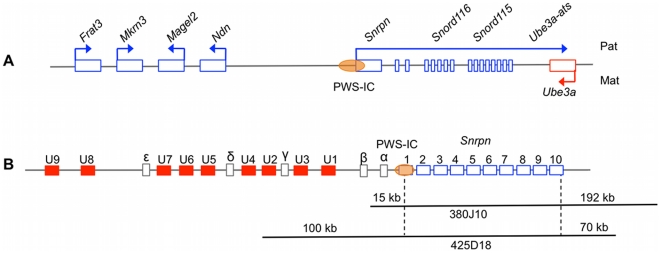
The PWS/AS imprinted domain and corresponding regions covered by the BAC transgenes. (A) Schematic diagram of the murine PWS/AS locus. Genes expressed from the paternal allele are shown above the locus (blue rectangles) and maternally expressed gene are shown below (red rectangles)(not to scale). (B) Schematic diagram of the *Snrpn* locus and sequences included in the 380J10 and 425D18 BACs. The nine identified *Snrpn* U exons (filled red boxes) are shown as well as five additional exons, termed α-ε (open boxes) [17]. For each BAC, the extent of genomic sequence relative to *Snrpn* is indicated (not to scale).

The PWS/AS locus is highly conserved from human to mouse in both gene order and allelic gene expression patterns, providing an excellent model for studying IC-directed imprinting mechanisms within this domain (Figure 1A). Hindering these studies has been the absence of an identifiable murine AS-IC. However, as in the human, the murine *Snrpn* locus contains several alternative upstream promoters and exons, herein referred to as U exons, from which multiple alternatively spliced transcripts arise [16], [17]. These U exons are transcribed exclusively from the paternal allele in the brain as well as in oocytes [15], [16], [18]. U exon expression in the oocyte suggests that transcription is essential for maternal imprinting at the PWS/AS locus. In this report, we test whether the murine U exons have AS-IC activity.

Previous targeted deletion approaches to knockout AS-IC function proved unsuccessful or resulted in only partial imprinting defects [19], [20], suggesting that the murine AS-IC consists of multiple elements. We have therefore taken a transgenic approach to identify the murine AS-IC. We utilized a BAC transgene that contains *Snrpn* and 100 kb of upstream sequence, including three of the alternative U exons. We show that this transgene is properly imprinted in multiple transgenic lines, indicating that there is a functional AS-IC contained within its sequence. Transcription of the U exons from the transgene correlates with imprint establishment in the maternal germ line. Upon targeted deletion of the three U exons, this transgene loses both the epigenetic imprint as well as its imprinted expression pattern, demonstrating that the AS-IC activity originates from the U exons.

## Results

### AS-IC Activity Is Contained within a 100-kb Region Upstream of *Snrpn*

**Figure 2:**
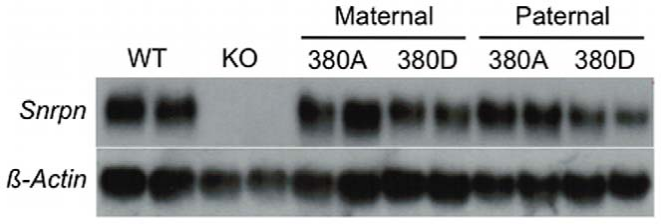
380J10 BAC transgene expression analysis. Northern blot analysis for *Snrpn* expression in the 380J10 transgenic lines. Two independent P1 brain samples were analyzed for each line and parental transmission. The PWS-IC^Δ35kb^ (KO) samples demonstrate the absence of endogenous *Snrpn* expression upon paternal transmission of this allele. The 380 transgenic samples all bear a paternal PWS-IC^Δ35kb^ allele. β-*actin* was assayed as a loading control.

To establish a transgenic system that would faithfully model AS-IC activity, it was necessary to identify a murine BAC that contained the PWS-IC and expressed *Snrpn* exclusively after paternal transmission. In addition, to study the role of the U exons, it was important to compare BAC transgenes containing U exons to those without. We screened the C57BL/6 RPCI-23 murine BAC library and identified BACs 380J10 and 425D18 for further study. The 380J10 BAC contains approximately 15 kb of sequence upstream of *Snrpn* exon 1, a region lacking any alternative *Snrpn* U exons. The 425D18 BAC possesses approximately 100 kb of sequence upstream of *Snrpn*, including three of the nine identified U exons (Figure 1B). We generated two independent transgenic lines for the 380J10 BAC, 380A and 380D. 380A contains multiple copies of the transgene while 380D is a single copy line (Figure S1A). We examined the imprinted status of the transgene by Northern blot analysis on postnatal (P) day 1 brain RNA. As the endogenous *Snrpn* transcript would complicate transgene expression analysis, we initially crossed our transgenic lines to a mutant bearing a 35 kb deletion of the PWS-IC (PWS-IC^Δ35kb^) [21]. This deletion ablates endogenous *Snrpn* expression when inherited paternally. The 380A and 380D lines were analyzed upon both maternal and paternal transmission of the transgene in combination with a paternal PWS-IC^Δ35kb^ allele. In both lines, transgene-encoded *Snrpn* was expressed after maternal and paternal transmission (Figure 2A). This result demonstrated that AS-IC activity is not contained within 15 kb upstream of *Snrpn*, a region deficient in U exons.

**Figure 3:**
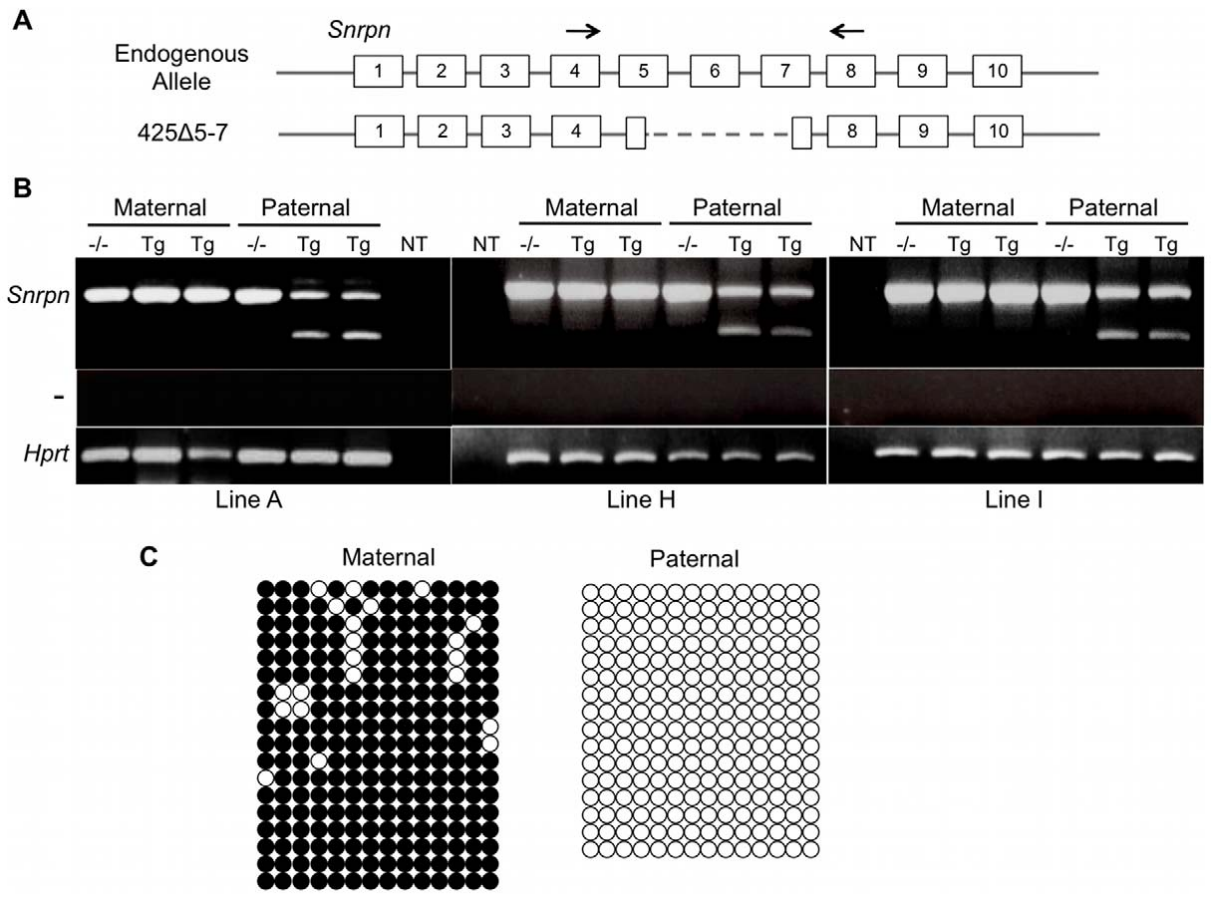
Analysis of imprinting for the 425Δ5-7 BAC transgene. (A) Schematic diagram of the strategy for *Snrpn* expression analysis by RT-PCR. The *Snrpn* gene contains ten exons (numbered boxes). The 425Δ5-7 transgene bears a deletion between exons 5 and 7 (dashed lines). PCR primers (arrows) in exons flanking the deletion distinguish the endogenous *Snrpn* product (513 bp) from transgenic *Snrpn* expression (350 bp). (B) RT-PCR analysis of P1 brain RNA from the 425Δ5-7A, 425Δ5-7H, and 425Δ5-7I (from left to right) transgenic lines. Non-transgenic littermates (-/-) were included as negative controls. NT indicates a control PCR reaction with no cDNA template added. *Hprt* amplification was performed to demonstrate cDNA integrity. The minus sign in RT-PCR analyses indicates control samples in which reverse transcriptase was omitted during cDNA synthesis. (C) Analysis of the DNA methylation imprint at the 425Δ5-7A *Snrpn* DMR. Genomic bisulfite sequencing was performed on P1 brain samples after both maternal and paternal transmission of the transgene. A 364 bp region of the *Snrpn* DMR (spanning from −175 to +189 relative to *Snrpn* exon 1) that contains 14 CpG dinucleotides [19] was analyzed in the 425Δ5-7A line. A SNP located within the sequenced region distinguishes the transgenic from the endogenous alleles. Each row represents an individually sequenced clone, filled circles indicate methylated CpG dinucleotides, and open circles represent unmethylated CpG dinucleotides. DNA methylation patterns at the endogenous locus are shown in Figure S2.

We then focused on the 425D18 BAC which contains three *Snrpn* U exons. To simplify transgene expression analysis, we utilized BAC recombineering techniques to delete a region between *Snrpn* exons 5 and 7, sequence shown previously to have no effect on imprinting at the locus [21]. We established multiple transgenic lines using this BAC, termed BAC 425Δ5-7. RT-PCR with primers located in exons 4 and 8, the exons flanking the deletion, distinguishes expression of endogenous *Snrpn* from expression of the transgene based on amplicon size (Figure 3A). Analysis of P1 brain RNA showed the 425Δ5-7 transgene was expressed upon paternal but not maternal inheritance and thus was correctly imprinted (Figure 3B). This result was demonstrated in three independent lines, lines A, H, and I, indicating that AS-IC activity lies within the 425D18 BAC sequence. We chose line 425Δ5-7A for further analysis as we found this line to be single copy (Figure S1B).

We further analyzed imprinting of the 425Δ5-7 transgene by examining the *Snrpn* differentially methylated region (DMR), which is located within the PWS-IC. In somatic cells, this DMR is methylated on the maternal chromosome and unmethylated on the paternal chromosome [22]–[24]. This DNA methylation imprint is erased in fetal germ cells and applied as a maternal-specific epigenetic mark during oogenesis [25]–[27]. To investigate whether the 425Δ5-7 BAC transgene undergoes appropriate epigenetic reprogramming in the germ line, we used genomic bisulfite sequence analysis to examine the methylation status of the *Snrpn* DMR in transgenic P1 brain DNA. We bred the 425Δ5-7A transgene onto a C57BL/6 line congenic for the PWS/AS domain of *Mus musculus castaneus* chromosome 7 (B6.*cast*.c7) [28] and examined a region within the DMR containing 14 CpG dinucleotides. A single nucleotide polymorphism (SNP) located within our region of interest distinguishes C57BL/6 transgenic from endogenous *cast* sequences. As expected, the paternally transmitted 425Δ5-7A transgene displayed hypomethylation at the *Snrpn* DMR while the maternally transmitted transgene was hypermethylated, demonstrating the presence of the appropriate epigenetic imprinting marks (Figure 3C). The endogenous alleles displayed the expected mixture of methylated and unmethylated clones as both the maternal and paternal alleles are represented in these sequences (Figure S2). Taken together, the expression analysis and DNA methylation analysis illustrate that the 425D18 BAC harbors complete AS-IC activity.

### *Snrpn* U Exon Expression Patterns Are Conserved on the 425Δ5-7 Transgene

The human AS-IC lies within a region of 880 bp that contains two of several alternative upstream exons of *SNRPN*, designated u5 and u6 [11], [13], [14], [29], [30]. These two exons are included in various transcripts originating from the upstream exons, suggesting a potential role in the imprinting process. A conserved sequence from the human AS-IC has not been identified in the mouse but the murine *Snprn* locus does possess a number of alternative U exons. Nine have been identified to date, termed U1-U9, spanning over 580 kb upstream of *Snrpn* exon 1 [17]. These exons generate multiple transcripts, most frequently splicing into exon 2 of *Snrpn* but occasionally splicing downstream of the *Snrpn* gene. Significantly, while *Snrpn* is widely expressed in adult tissues, U exon expression is detected only in postnatal brain from the paternal allele as well as in oocytes [15], [16], [18]. Expression of the U exons in oocytes correlates with imprint establishment in the female germ line, consistent with a role in establishing the maternal epigenotype.

**Figure 4:**
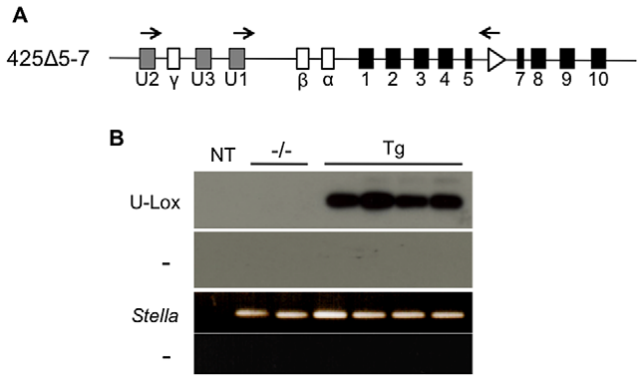
U exon expression from the 425Δ5-7A transgene during maternal imprint establishment. (A) Schematic diagram of the RT-PCR strategy used to analyze U exon transcription from the 425Δ5-7A transgene. The *lox*P site, unique to the transgene exon 5 to exon 7 deletion, is depicted as an open triangle. PCR primers (arrows) were designed to anneal to U1 or U2 and the *lox*P site. (B) RT-PCR analysis of U exon expression in ovary RNA. Two wild type (−/−) and four transgenic (Tg) sets of ovaries were analyzed from females at three weeks of age. Transgenes were maternally transmitted. *Stella* amplification was performed to verify the presence of oocyte RNA.

As the 425Δ5-7A transgene displays appropriate imprinted expression and epigenetic imprinting marks, we hypothesized that U exon expression from this transgene would mimic the endogenous U exon expression patterns. To detect U exon transcripts arising exclusively from the transgene, we used a primer recognizing a *lox*P site that remained in the BAC after deletion of the region between *Snrpn* exons 5 and 7. We performed RT-PCR using a forward primer that anneals to both U1 and U2 (referred to as U-F1) and a reverse primer in the *lox*P sequence (Figure 4A). Replicating the endogenous locus, BAC transgene-encoded transcripts including the U exons were detected in P1 brain after paternal transmission of the transgene but not after maternal transmission (Figure S3). Importantly, these transcripts were also detected in transgenic ovary RNA from three-week-old females, corresponding with the timing of imprint establishment in oocytes (Figure 4B). Thus, U exon expression from the imprinted 425Δ5-7A transgene does mimic the endogenous expression pattern.

### *Snrpn* U Exon Expression in the Germ Line Correlates with Maternal Imprint Establishment

**Figure 5:**
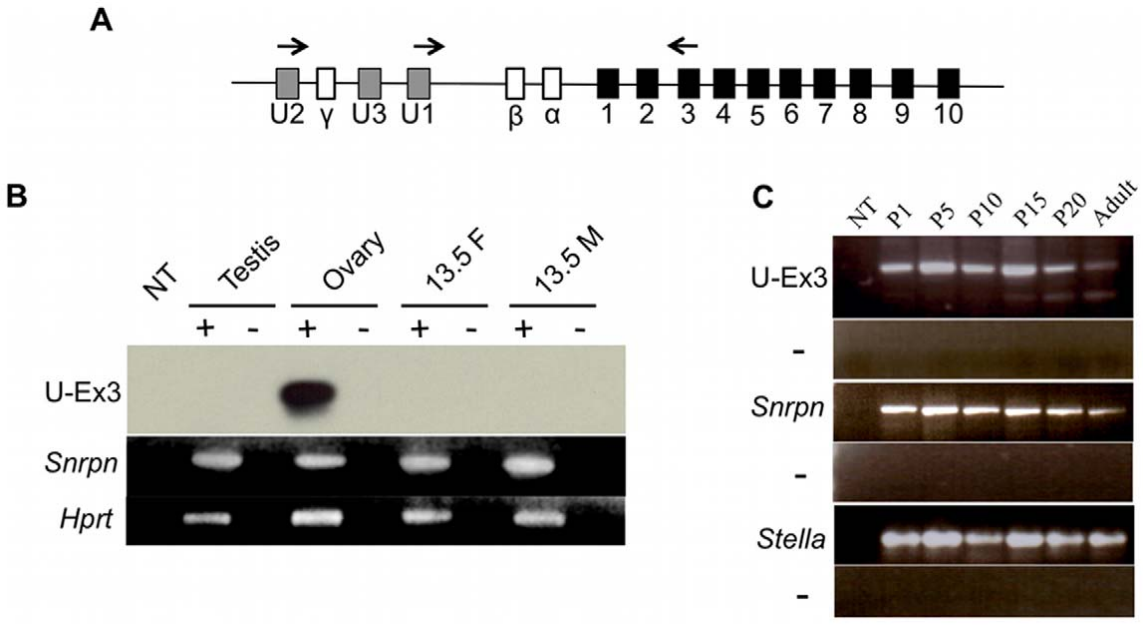
U exon expression in the developing germ line. (A) Schematic diagram of the RT-PCR strategy used to analyze U exon transcription. PCR primers (arrows) were designed to anneal to U1 or U2 and *Snrpn* exon 3 [16]. (B) RT-PCR analysis of U exon expression in 13.5 dpc PGCs from female (F) and male (M) embryos. Testis and adult ovary provide negative and positive controls, respectively. The RT-PCR reactions were Southern blotted and probed with verified product to confirm identity. *Snrpn* expression was also analyzed using primers spanning exons 4 to 8. (C) U exon expression was analyzed by RT-PCR in wild type ovaries spanning from P1 to adult. The identity of the products was confirmed by DNA sequencing. *Stella* expression was examined to verify the presence of oocyte RNA.

We explored the role of *Snrpn* U exon transcription in directing imprinting at the PWS/AS locus by analyzing U exon expression in the developing germ line. Imprints are erased in the germ line in post-migratory germ cells between 10.5 to 12.5 days post coitus (dpc), yielding biallelic expression of *Snrpn* in 13.5 dpc primordial germ cells (PGCs) [27], [31]–[34]. Therefore, we examined 13.5 dpc PGCs for the presence of *Snrpn* U exon-containing transcripts, expecting a lack of expression since imprinting is not established until after birth in the maternal germ line. We utilized the U-F1 primer and a reverse primer complementary to *Snrpn* exon 3 to analyze expression in wild type, sex-segregated, purified 13.5 dpc PGC cDNAs (Figure 5A). *Snrpn* U exon-containing transcripts were not detected in either male or female 13.5 dpc PGCs (Figure 5B). RT-PCR was performed on the same cDNAs using the primer set spanning *Snrpn* exons 4 to 8 to demonstrate that *Snrpn* is expressed in both male and female 13.5 dpc PGCs. These data confirm that U exon transcription is not detectable in the germ line before imprints are established.

We further tested whether U exon expression contributed to imprinting by examining the postnatal ovary. Detecting expression in this tissue is vital as the DNA methylation imprint is established at the PWS-IC in growing oocytes. The *Snrpn* DMR methylation imprint first appears in growing oocytes between 5–25 days postpartum (dpp) [25], [26]. Therefore, we examined ovaries from wild type female mice to determine whether U exon transcription was active throughout this stage of imprint establishment. Ovaries obtained from females ranging in age from P1 to P20 were subjected RT-PCR analysis for transcripts spanning U-F1 to *Snrpn* exon 3. We detected U exon-containing transcripts in the ovary during the entire period of maternal imprint establishment at the PWS-IC (Figure 5C). This RT-PCR reaction revealed a larger amplicon than expected that was preferentially expressed in the developing ovaries. DNA sequencing of the PCR products indicated that the larger amplicon was derived from a transcript originating at U2 and included the *Snrpn* γ exon whereas the smaller product included only U1 and *Snrpn* exons 2 and 3.

### Three *Snrpn* U Exons Constitute the AS-IC on the 425D18 BAC Transgene

**Figure 6:**
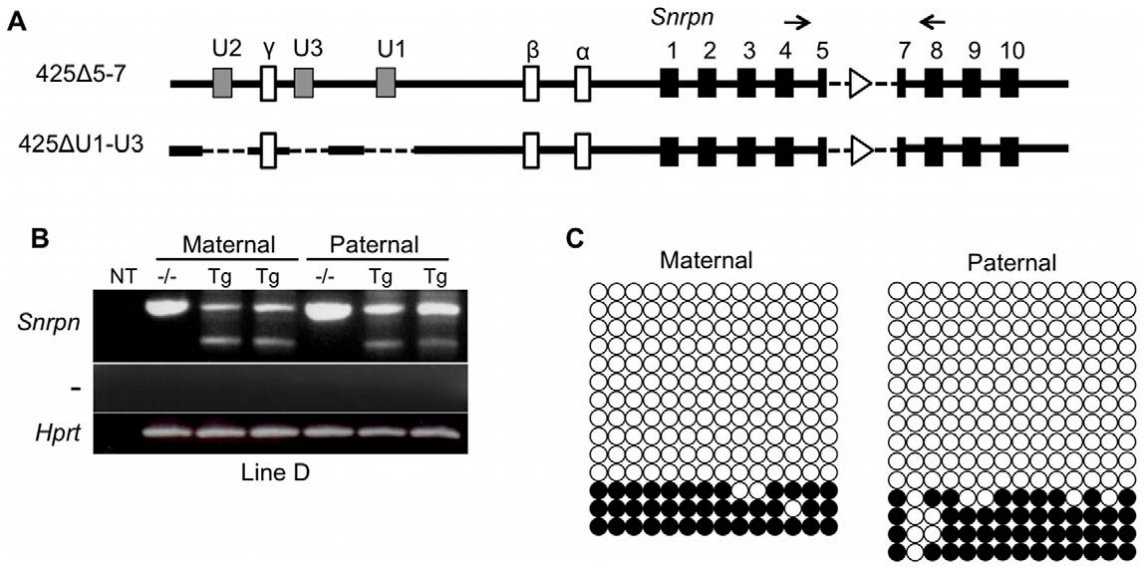
Analysis of imprinting of the 425ΔU1-U3D BAC transgene. (A) Schematic representation of the 425ΔU1-U3 transgene. The three upstream exons (gray boxes) were deleted (dashed lines) from the 425Δ5-7 BAC to create this transgene. PCR primers (arrows) in exons flanking the deletion distinguish the endogenous *Snrpn* product (513 bp) from transgenic *Snrpn* expression (350 bp). (B) Expression analysis of the 425ΔU1-U3D transgene. RT-PCR was performed on P1 brain RNA after both maternal and paternal transmission of the transgene. (C) Analysis of the DNA methylation imprint at the 425ΔU1-U3D *Snrpn* DMR. Genomic bisulfite sequencing was performed on P1 brain samples after both maternal and paternal transmission of the transgene. A SNP located within the sequenced region distinguishes the transgenic from the endogenous alleles, only the transgenic alleles are shown.

We next sought to determine whether the *Snrpn* U exons are necessary for maternal imprinting of the 425Δ5-7 transgene. To do so, we used BAC recombineering to make targeted deletions of U1, U2, and U3, the three U exons contained within the 425Δ5-7 transgene. For each U exon, a 2.5 to 2.7 kb region spanning the exon was deleted to eliminate promoter function. This modified BAC was termed 425ΔU1-U3 (Figure 6A). Transgene copy number is an important element in imprinted transgene studies, especially as a multi-copy *Snrpn* transgene was previously shown to be imprinted while the same transgene in single copy was biparentally expressed [35]. We therefore limited our analysis to single copy 425ΔU1-U3 transgenic lines. In addition to providing the most stringent test of imprinting, single copy transgene lines eliminate uncertainty about which copy(s) of the transgene is being expressed and allow for accurate determination of DNA methylation status. The single copy line 425ΔU1-U3D was used for our initial analysis (Figure S1B). Unlike the 425Δ5-7 transgene, the 425ΔU1-U3D transgene was expressed in P1 brain upon both paternal and maternal inheritance, indicating an absence of AS-IC activity correlating with the deletion of the upstream exons (Figure 6B). We also examined the methylation imprint at the transgene *Snrpn* DMR and found that it was hypomethylated after both paternal and maternal transmission, demonstrating an absence of the maternal epigenetic imprint (Figure 6C). These experiments show that the 425Δ5-7 transgene loses its imprinted expression pattern as well as its epigenetic imprint upon removal of the three U exons contained within it, providing further evidence that these U exons play a major role in AS-IC function.

### Imprinting Is Established in the Absence of the U Exons when Transcription through the PWS-IC Is Activated

**Figure 7:**
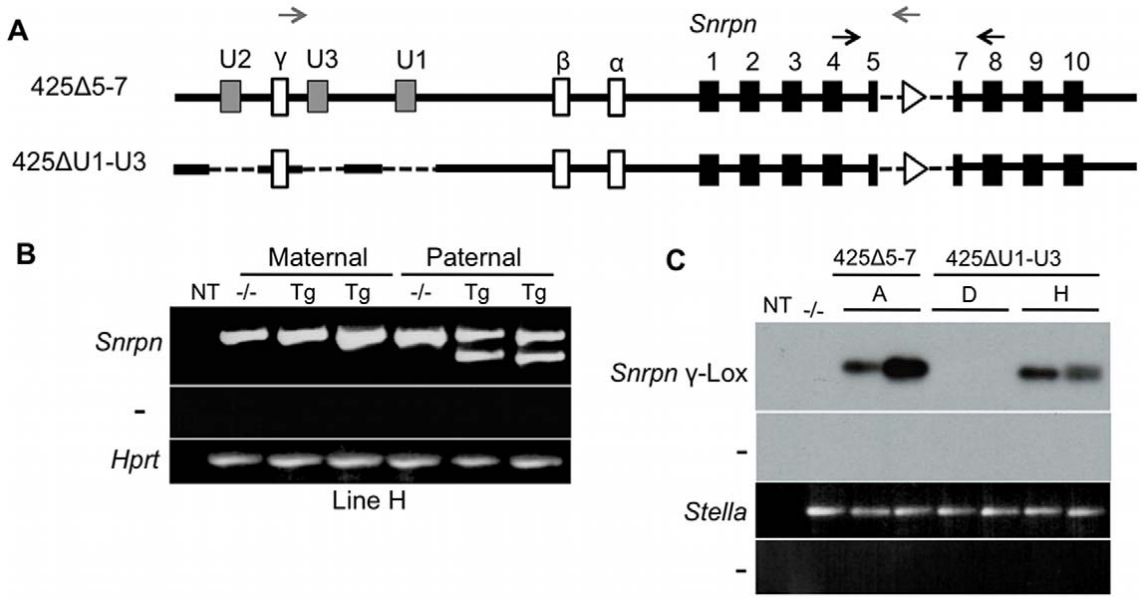
Expression analysis of the imprinted 425ΔU1-U3H transgene. (A) Schematic representation of the 425ΔU1-U3 transgene. PCR primers (black arrows) in exons flanking the deletion distinguish the endogenous *Snrpn* product (513 bp) from transgenic *Snrpn* expression (350 bp). (B) Expression analysis of the 425ΔU1-U3H transgene. RT-PCR analysis of P1 brain RNA after both maternal and paternal transmission of the transgene. (C) Analysis of transcription across the PWS-IC in transgenic ovaries during imprint establishment. P15 ovaries were isolated from 425Δ5-7A, 425ΔU1-U3D, and 425ΔU1-U3H females and analyzed by RT-PCR for *Snrpn* γ exon-containing transcripts. Primers anneal to the *Snrpn* γ exon and the *lox*P site (gray arrows in (A)). The RT-PCR product was Southern blotted and probed with verified product to confirm identity. *Stella* expression was examined to demonstrate the presence of oocyte RNA.

We obtained one additional single copy line for the 425ΔU1-U3 transgene, 425ΔU1-U3H (Figure S1B). Unexpectedly, this transgene displayed imprinted expression of *Snrpn* (Figure 7B). We initially questioned whether critical sequences in the BAC had been rearranged upon integration but found that sequences for all single copy BAC transgenes were intact between U1 and the PWS-IC (Figure S4). Since the insertion sites for the transgenes in our study are unknown, we questioned whether transcripts initiated from a promoter upstream of the 425ΔU1-U3H transgene insertion site might proceed through the PWS-IC in the maternal germ line. We reasoned that this adventitious transcription could create an artificial imprint at the PWS-IC. To investigate this hypothesis, we performed RT-PCR on ovaries harvested from P15 females, including the imprinted 425ΔU1-U3H and 425Δ5-7A lines as well as the non-imprinted 425ΔU1-U3D line. As the 425ΔU1-U3 transgenes do not have the U exons intact, we used sequence from the *Snrpn* γ exon to generate our forward primer for RT-PCR since this exon is included in U exon transcripts generated during oocyte growth (Figure 5C). Unlike U1-U9, the Greek-lettered exons shown in Figure 1B have both splice acceptor signals in addition to splice donor signals [17]. We reasoned that the γ exon could be included in transcripts initiated from upstream of the transgene insertion site. As before, the reverse primer in the *lox*P sequence was used so as to avoid any signal from the endogenous allele. Figure 7C demonstrates that ovaries from the imprinted 425ΔU1-U3H line expressed *Snrpn* γ exon-containing transcripts that traversed the PWS-IC. These transcripts were also observed in the imprinted 425Δ5-7A line but not in the biparentally expressed 425ΔU1-U3D line. Thus, transcription through the PWS-IC in the ovary correlates with the establishment of the maternal imprint.

## Discussion

Roughly 4% of individuals with AS have an imprinting defect, characterized by a paternal epigenotype at the PWS/AS locus on the maternally inherited chromosome. Of these individuals, 10–15% have a microdeletion that includes the AS-IC [36]. The AS-IC is thought to operate in the female germ line to epigenetically inactivate the PWS-IC and thereby silence the paternally expressed genes on the future maternal allele [9]. Among these maternally silenced genes is *UBE3A-ATS*, a transcript antisense to *UBE3A*. *UBE3A-ATS* is thought to silence *UBE3A* expression by an unknown mechanism [37]–[39]. In individuals lacking AS-IC function, *UBE3A-ATS* is expressed from the maternal allele in addition to the paternal allele and the consequent lack of *UBE3A* results in AS. Hypomethylation of the maternal *SNRPN* DMR is diagnostic of an AS imprinting defect [40].

The AS-IC functions in the female germ line, complicating investigations into imprinting mechanisms at the PWS/AS locus. Deletion analysis mapped the human AS-IC to 35 kb upstream of the PWS-IC but deletion of similarly positioned sequences in the mouse had no detectable consequence [19]. Here we used BAC transgenes to identify murine AS-IC activity. We found that 15 kb of sequence upstream of *Snrpn* exon 1 was insufficient to direct imprinted expression of *Snrpn*. Transgenes containing 100 kb of sequence 5′ to *Snrpn* exon 1 directed the appropriate imprinted expression of *Snrpn*. Furthermore, this sequence was sufficient to direct appropriate hypermethylation of the *Snrpn* DMR following maternal but not paternal transmission. We conclude that an AS-IC activity is contained within 15 to 100 kb of the PWS-IC. This region contains three alternative exons, at least two of which are expressed in oocytes and splice into *Snrpn* exon 2. Deletion of these U exons from the BAC transgene resulted in biparental *Snrpn* expression and DNA hypomethylation at the PWS-IC, characteristic of an imprinting defect. Surprisingly, a second single copy line lacking the U exons displayed appropriate maternal silencing. In contrast to the biparentally expressed transgene but similar to imprinted transgenes with the U exons intact, this line exhibited transcription through the PWS-IC, consistent with the notion that transcription is an essential element of AS-IC activity. Lastly, we demonstrated that U exon transcription is undetectable in fetal oogonia as they are entering meiotic prophase, a time when imprinting is absent, but becomes evident after birth and prior to the application of the DNA methylation imprint at the PWS-IC. We conclude that the U exons exhibit AS-IC activity as previously proposed [8], [15].

**Figure 8:**
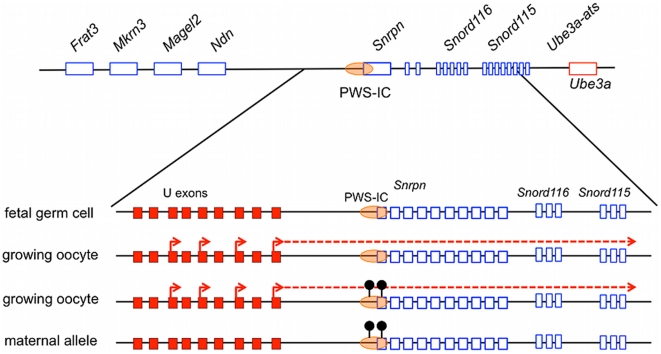
A working model for the establishment of the maternal imprint at the PWS-IC. Schematic diagrams of the locus around *Snrpn* at various stages of development. The U exons are indicated by red boxes and the PWS-IC is depicted as an orange oval overlying *Snrpn* exon 1. Imprints are erased in fetal germ cells and the *Snrpn* upstream exons are not expressed. In the neonatal period, transcription is initiated from the *Snrpn* upstream exons prior to the appearance of the DNA methylation imprint in growing oocytes. These transcripts proceed through the PWS-IC, triggering repressive epigenetic modification of this element, indicated by black lollipops. The PWS-IC on the future maternal allele is thereby inactivated, rendering the paternally expressed genes silent.

*Snrpn* transcription becomes biallelic in PGCs as they colonize the gonad at midgestation [31]. This stage of imprinting erasure in the germ line coincides with the removal of the DNA methylation imprint at the PWS-IC [27]. The appearance of the DNA methylation imprint is tied to postnatal oocyte growth [25], [26]. Our data support a model in which growing oocytes direct transcription from the U exons through the PWS-IC. This transcription is necessary to epigenetically modify the PWS-IC leading to the maternal imprint (Figure 8). Importantly, our results do not distinguish whether the RNA transcript or the act of transcription is essential for AS-IC activity. However, since transcription presumably arising from sequences flanking the BAC insertion site appears to be sufficient for imprinting in the 425ΔU1-U3H line, we suggest that the U exon sequences *per se* are not required.

Our data support a model in which U exon promoter activity supplies AS-IC function in the mouse. The human AS-IC, however, includes two upstream exons that are internal to transcripts encoded in brain RNA. If the human AS-IC does not possess promoter activity in oocytes, we speculate that the AS-IC may serve an RNA antitermination or stabilization role.

In both human and mouse, splicing of the upstream exons to *Snrpn* exon 2 can encode the Snurf polypeptide [17], [41]. It is unknown whether *Snurf* is expressed in oocytes but the absence of an overt phenotype in *Snurf* knockout mice indicates that it is not involved in the imprinting process [42].

Our BAC transgene system allows simultaneous assessment of both the transgene and endogenous locus. U exon transcription arising from the endogenous locus does not impose imprinting upon a maternally transmitted transgene harboring a PWS-IC but lacking U exons (Figure 2 and Figure 6B). This observation suggests that if the RNA transcript is an essential element of the imprinting machinery, then it does not function in *trans*.

Wu *et al.* [20] described two mutants with a maternal imprinting defect. Maternal transmission of an 80 kb deletion spanning from 10 to 90 kb upstream of *Snrpn* exon 1 resulted in an extensive loss of DNA methylation at the PWS-IC in only three of eight offspring. This deletion removes only U1 and U3, leaving the remaining seven U exons intact. Our model suggests that extensive imprinting defects were not observed in the remaining offspring due to compensatory transcription arising from the remaining U exons. The second mutant described by these investigators exhibited a highly penetrant imprinting defect leading to expression of paternal genes from the maternal allele and reduced expression of the *Ube3a* gene product, E6AP. This mutant carried an insertion 10 kb upstream of *Snrpn* exon 1 that was comprised of a *puromycin* resistance cassette in the opposite transcriptional orientation to *Snrpn*, exons 3–9 of an *Hprt* minigene, and a duplication of 6 kb of endogenous sequence. Our findings support a model in which the insertion interferes with transcription from the U exons in oocytes.

The imprinting activity of the human AS-IC element has been tested in mice. Five independent lines of a human P1 phage transgene that included both the human AS-IC and PWS-IC failed to show evidence of imprinted gene expression in the brain [35]. This lack of imprinting may be explained by our more recent observation that a human PWS-IC substituted for the endogenous murine PWS-IC can acquire a DNA methylation imprint in oocytes but this imprint is subsequently lost during development [38]. Shemer *et al.* [10] characterized transgenes containing the human AS-IC linked to the murine PWS-IC and observed preferential *Snrpn* expression following paternal inheritance. Transcription through the PWS-IC during oogenesis was not examined in these transgenes but our model suggests that the partial imprinting seen in these transgenes resulted from transcription of the human upstream exons.

Zogel *et al.* [43] reported that a maternally inherited polymorphism at the AS-IC increases the risk of imprinting defects leading to AS. Our observations strongly support these investigators′ hypothesis that this polymorphism may affect a transcription factor recognition site. Our model suggests that this polymorphism affects transcription through the PWS-IC during oogenesis, decreasing the efficiency of epigenetic modification at the PWS-IC and thereby raising the risk of AS imprinting defects.

Transcription is implicated in allelic silencing at several imprinted gene clusters (See Peters and Robson [44] for a review). Our results at the PWS/AS locus are consistent with the observation that in growing oocytes, transcripts traverse maternal germ line DMRs at eight locations, suggesting that transcription is commonly associated with the establishment of maternal imprints [45]. A recent report indicates that 35% of CpG islands that are methylated in oocytes are downstream of active promoters [46]. The role of transcription through a germ line DMR in establishing a DNA methylation imprint in oocytes has been tested at the *Gnas* locus [45]. At this locus, *Nesp* transcription is active in oocytes and traverses two maternal DMRs. Similar to the U transcripts at the PWS/AS locus, appearance of the *Nesp* transcript at the *Gnas* locus temporally precedes the establishment of the maternal methylation imprint in growing oocytes. Furthermore, truncation of the *Nesp* transcript led to a loss of the DNA methylation imprint at several maternal DMRs within the *Gnas* locus and activation of normally silent maternal transcripts. Chotalia *et al.* [45] suggested several mechanisms by which transcription might contribute to DNA methylation including an interaction of the transcription complex with the methylation machinery, creating an open chromatin environment allowing DNA methylation machinery access to the locus, or recruiting factors necessary for epigenetic modification. These same mechanisms could operate to set the methylation imprint at the PWS/AS locus. Similar to the *Gnas* locus, truncation of the U exon transcripts upstream of the PWS-IC may confirm their role in establishing imprinting throughout the PWS/AS locus.

## Materials and Methods

### Ethics Statement

All animal procedures in this work were reviewed and approved by the University of Florida Institutional Animal Care and Use Committee.

### BAC DNAs

BACs 380J10 and 425D18 were originally identified by application of a *Snrpn* probe to the RPCI-23 library prepared from C57BL/6 DNA (Research Genetics). BAC 380J10 is 232.3 kb and contains chromosome 7 nucleotides (nt) 67165323 to 66935240. This region includes 15.3 kb upstream of the major *Snrpn* transcription start site to 192.1 kb 3′ sequence containing nearly the entire complement of *Snord116* repeats. BAC 425D18 is 192.3 kb and contains sequences from nt 67250191 to 67057909. This span includes 100.1 kb upstream of the major *Snrpn* transcription start site and 69.5 kb sequence 3′ to the *Snrpn* polyadenylation site.

Recombineering procedures were performed in bacterial strains DY380, EL250 and EL350 as described [47]. First, a *lox*P site in the BACe3.6 vector was replaced with a *blasticidin* resistance cassette. To create a reporter gene in the BAC, a deletion spanning *Snrpn* exons 5 to 7 was engineered by homologous recombination with a floxed *kanamycin* (*kan*) resistance cassette followed by Cre-mediated removal of the *kan* resistance cassette in EL350 cells. This deletion removes sequences previously shown to be unimportant for imprinting [21]. The three U exons in this modified BAC were deleted individually by first subcloning a 4.6 to 5.0 kb region surrounding each U exon and subsequently inserting a *kan* resistance cassette into each clone. These clones were then used to recombineer the modified 425D18 BAC. The U1 deletion removes 984 bp 5′ to 1576 bp 3′ of U1. The U2 deletion removes sequences from 1032 bp 5′ to 1296 bp 3′ of U2. The U3 deletion removes sequences from 834 bp 5′ to 1575 bp 3′ of U3. In each case, the *kan* resistance cassette was removed by recombineering. Recombination between *lox* sites at different U exons was prevented by using *kan* resistance cassettes flanked by FRT sites at U1 and by incompatible *lox* sequences at U2 and U3 [48]. Transfer of each deletion to the BAC was confirmed by Southern blot analysis and DNA sequencing.

### Transgenic Mice

C57BL/6 BAC DNA was purified by CsCl gradient centrifugation and supercoiled DNA was injected into fertilized FVB/N oocytes. Strains were maintained on an FVB background except where noted for the DNA methylation studies.

### Southern Blot Analysis

Genomic DNA was digested with restriction endonucleases as indicated and analyzed by Southern blot. The 380J10 copy number blot was probed with a 770 bp *EcoR*I fragment approximately 7.8 kb upstream of *Snrpn* exon 1. The 425D18 copy number blot was probed with a 291 bp *EcoR*I fragment located 5′ to the deletion between *Snrpn* exons 5 and 7. Relative band intensity was digitized on the Storm 860 PhosphorImager (GE Healthcare) and analyzed with Image Quant TL software for transgene copy number analyses. Probes for Southern blots demonstrating the integrity of the 425D18 derived BAC transgenes were generated from PCR products (H, E,L) (see Dataset S1 for primer sequences) or restriction fragments (304: 800 bp *Pst*I fragment, 210: 770 bp *EcoR*I fragment).

### Analysis of Gene Expression

RNA was isolated from the indicated tissues by homogenization with RNA-Bee reagent (Tel-Test, Inc.) following manufacturer's instructions. RNA was subject to Northern blot analysis or DNase treated and subsequently reverse-transcribed with Superscript II (Invitrogen). PCR reactions were performed under standard conditions. Primer sequences listed in Dataset S1. The *Snrpn* Northern blot probe was generated from a 480 bp *Cla*I-*Pst*I fragment.

### DNA Methylation Analysis

Bisulfite sequence analysis was performed on whole brain genomic DNA. DNA was subject to bisulfite conversion as previously described [49]. PCR amplification of the *Snrpn* DMR was performed on the bisulfite converted DNA using the primer set W18-F/W19-R [19] (Dataset S1). An initial 15 minute denaturation step was followed by 38 cycles of 94°C for 45 s, 54°C for 60 s and 72°C for 90 s with a 10 minute final extension at 72°C. Amplified products were cloned into the pGEM-T Easy Vector System (Promega). Plasmid sequencing was performed with ABI Prism BigDye terminator (PerkinElmer) at the UF Center for Epigenetics.

## Supporting Information

Dataset S1 PCR primer set sequences. The primers listed above were used for RT-PCR gene expression analysis, genomic bisulfite sequencing analysis, or Southern or Northern blot probe syntheses.
(DOC)

Figure S1 Transgene copy number analysis. Copy number was assessed by Southern blot analysis on genomic brain DNA following digestion with the indicated restriction endonucleases. Two samples from each transgenic line were analyzed and the ratio of the average transgenic to endogenous allele band intensity is displayed below the lanes. (A) Copy number Southern blot for the 380J10 transgenic lines. Genomic DNAs were digested with *Pst*I and hybridized with a probe for the 5′ end of the transgene. The endogenous allele generates a 17.8 kb fragment while the transgene produces a 12.5 kb fragment. (B) Copy number Southern blot for the 425D18 transgenic lines. Genomic DNAs were digested with *Spe*I and *Pvu*II and hybridized with a probe just 5′ to the deletion between *Snrpn* exons 5 and 7. The endogenous allele generates a 4.5 kb fragment while the transgene produces a 3.0 kb fragment.
(TIFF)

Figure S2 DNA methylation analysis of the *Snrpn* DMR. Genomic bisulfite sequencing was performed on P1 brain samples after both maternal and paternal transmission of the 425Δ5-7A transgene. The methylation status of the endogenous alleles is represented here. Each row represents an individually sequenced clone. Filled circles indicate methylated CpG dinucleotides and white circles represent unmethylated CpG dinucleotides.
(TIFF)

Figure S3 U exon usage from the 425Δ5-7A transgene in the brain. (A) Schematic diagram of the RT-PCR strategy used to analyze U exon transcription from the 425Δ5-7A transgene. The *lox*P site is depicted as a white triangle. PCR primers (arrows) were designed to anneal to U1 or U2 and the *lox*P site. (B) RT-PCR analysis of U exon expression was performed on P1 brain RNA after maternal and paternal transmission of the transgene. *Hprt* amplification was performed to demonstrate cDNA integrity.
(TIFF)

Figure S4 The single copy 425D18 derived BAC transgenes are intact and not rearranged. The topmost part of the figure shows 100 kb upstream of *Snrpn* exon 1. Black rectangles indicate the three U exons present in the 425D18 BAC as well as *Snrpn* exon 1. The locations of five probes, termed H, E, L, 304, and 210, are shown in red (arrows). Horizontal lines indicate the lengths and locations of expected fragments for each restriction endonuclease and probe combination. Below each line is the expected size of that fragment, first from the BAC containing U exons, 425Δ5-7, followed by the BAC from which the three U exons have been deleted, 425ΔU1-U3. Restriction endonuclease digests of either transgenic genomic DNA (lanes 1–3) or purified BAC DNA (lanes 4 & 5) were analyzed by Southern blot. Bands differing from the endogenous alleles are detectable only for fragments that include recombineered deletions of the U exons, indicating the absence of rearrangements within that region. Fragments overlap the entire region with the exception of a 1.4 kb span between the *Spe*I fragment recognized by probe E and the *Pst*I fragment recognized by probe L. A non-repetitive probe for this short region could not be identified. Lane 1: 425Δ5-7A genomic DNA, Lane 2: 425ΔU1-U3D genomic DNA, Lane 3: 425ΔU1-U3H genomic DNA, Lane 4: 425Δ5-7 BAC DNA, Lane 5: 425ΔU1-U3 BAC DNA.
(TIFF)
