# Supplementary material for: Transcription Is Required to Establish Maternal Imprinting at the Prader-Willi Syndrome and Angelman Syndrome Locus
Source: PLoS Genet. 2011 Dec 29;7(12):e1002422. doi: 10.1371/journal.pgen.1002422 (PMC3248558; doi:10.1371/journal.pgen.1002422)
Supplement: Dataset S1 — PCR primer set sequences. The primers listed above were used for RT-PCR gene expression analysis, genomic bisulfite sequencing analysis, or Southern or Northern blot probe syntheses. (DOC) [file pgen.1002422.s001.doc]

Data set S1

pcr primer set sequences

Table 1. List of primers used in RT-PCR experiments and for probe syntheses

| Primer name | Primer sequence (5’-3’) | Primer use |
| --- | --- | --- |
| Hprt-F | GCTGGTGAAAAGGACCTCT | Hprt |
| Hprt-R | CACAGGACTAGAACACCTGC | Hprt |
| N2.1-F | CCCCGAGTATTAAGGATCTTG | Snrpn Ex 4-8 |
| N6.2-R | GCAACAGTGCCTCTTCCCTG | Snrpn Ex 4-8 |
| SnrpnU-F1 | AAAGGAGCCTGACACATCCA | Snrpn U-Ex3, U-lox |
| SnrpnEx3-R | CCTTGAATTCCACCACCTTG | Snrpn U-Ex3 |
| Lox Tg-R2 | GATTTGTCCTACTCAGGAG | Snrpn U-lox, γ-lox |
| Snrpn γ-F3 | CTTGGAAGATAACCCACTG | Snrpn γ-lox |
| W18-F | GTAGTAGGAATGTTTAAGTATTTTTTTTGG | Snrpn DMR Bisulfite |
| W19-R | CCAATTCTCAAAAATAAAAATATCTAAATT | Snrpn DMR Bisulfite |
| Stella-F | TGAGTTTGAACGGGACAGTG | Stella |
| Stella-R2 | AGCTTTCACATCTGCTGTGC | Stella |
| β-actin-5’ | GTGGGCCGCTCTAGGCACCAA | β-actin Probe |
| β-actin-3’ | CTCTTTGATGTCACGCACGATTTC | β-actin Probe |
| 15.1-F | TACGACTTACCATATTATAC |   |
| 16.1-R | CACAACAATGGAGTGGAGGC | H Probe |
| G33-F1 | GAAGCAGTGGGTGAGTTTCC | E Probe |
| G34-R1 | TTATTCATAACCCCGTCTTTC | E Probe |
| U1del-F1 | GACTTCAGTACATGTTCCAAC | L Probe |
| 55.6-R | TGTGGGGCAGGGACTAGGAC | L Probe |
|  |  |  |
|  |  |  |
|  |  |  |
|  |  |  |
|  |  |  |
|  |  |  |
