# Supplementary figures and images for: Transcription Is Required to Establish Maternal Imprinting at the Prader-Willi Syndrome and Angelman Syndrome Locus
Source: PLoS Genet. 2011 Dec 29;7(12):e1002422. doi: 10.1371/journal.pgen.1002422 (PMC3248558; doi:10.1371/journal.pgen.1002422)

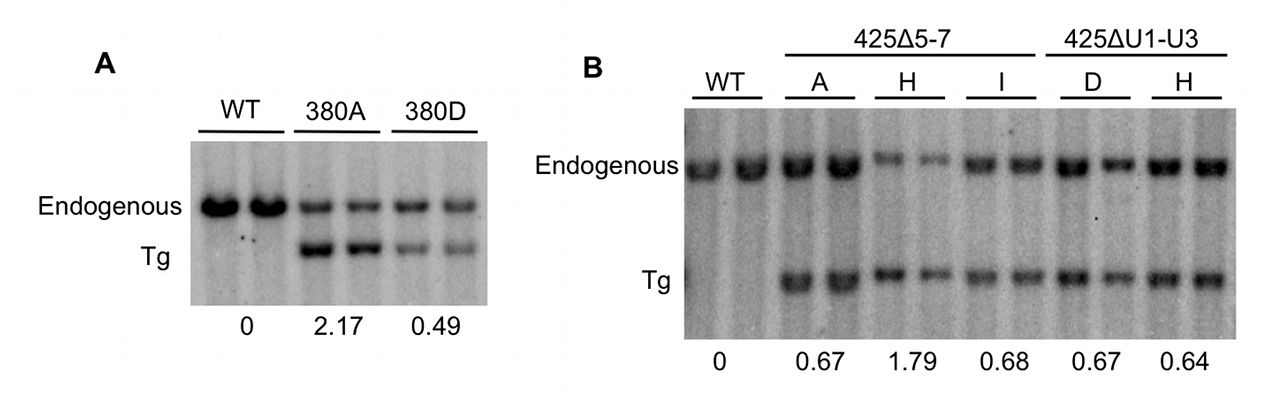

Supplement: Figure S1 — Transgene copy number analysis. Copy number was assessed by Southern blot analysis on genomic brain DNA following digestion with the indicated restriction endonucleases. Two samples from each transgenic line were analyzed and the ratio of the average transgenic to endogenous allele band intensity is displayed below the lanes. (A) Copy number Southern blot for the 380J10 transgenic lines. Genomic DNAs were digested with PstI and hybridized with a probe for the 5′ end of the transgene. The endogenous allele generates a 17.8 kb fragment while the transgene produces a 12.5 kb fragment. (B) Copy number Southern blot for the 425D18 transgenic lines. Genomic DNAs were digested with SpeI and PvuII and hybridized with a probe just 5′ to the deletion between Snrpn exons 5 and 7. The endogenous allele generates a 4.5 kb fragment while the transgene produces a 3.0 kb fragment. (TIFF) [file pgen.1002422.s002.tiff]

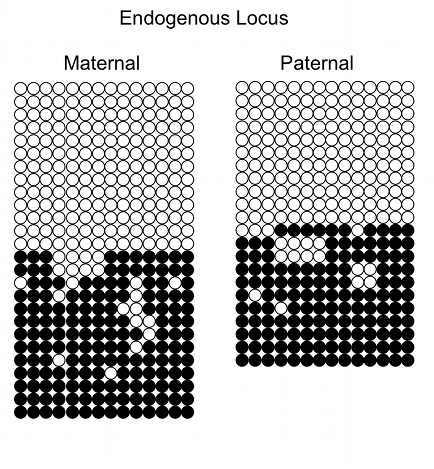

Supplement: Figure S2 — DNA methylation analysis of the Snrpn DMR. Genomic bisulfite sequencing was performed on P1 brain samples after both maternal and paternal transmission of the 425Δ5-7A transgene. The methylation status of the endogenous alleles is represented here. Each row represents an individually sequenced clone. Filled circles indicate methylated CpG dinucleotides and white circles represent unmethylated CpG dinucleotides. (TIFF) [file pgen.1002422.s003.tiff]

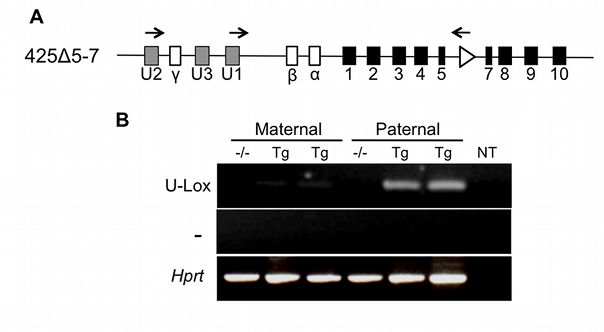

Supplement: Figure S3 — U exon usage from the 425Δ5-7A transgene in the brain. (A) Schematic diagram of the RT-PCR strategy used to analyze U exon transcription from the 425Δ5-7A transgene. The loxP site is depicted as a white triangle. PCR primers (arrows) were designed to anneal to U1 or U2 and the loxP site. (B) RT-PCR analysis of U exon expression was performed on P1 brain RNA after maternal and paternal transmission of the transgene. Hprt amplification was performed to demonstrate cDNA integrity. (TIFF) [file pgen.1002422.s004.tiff]

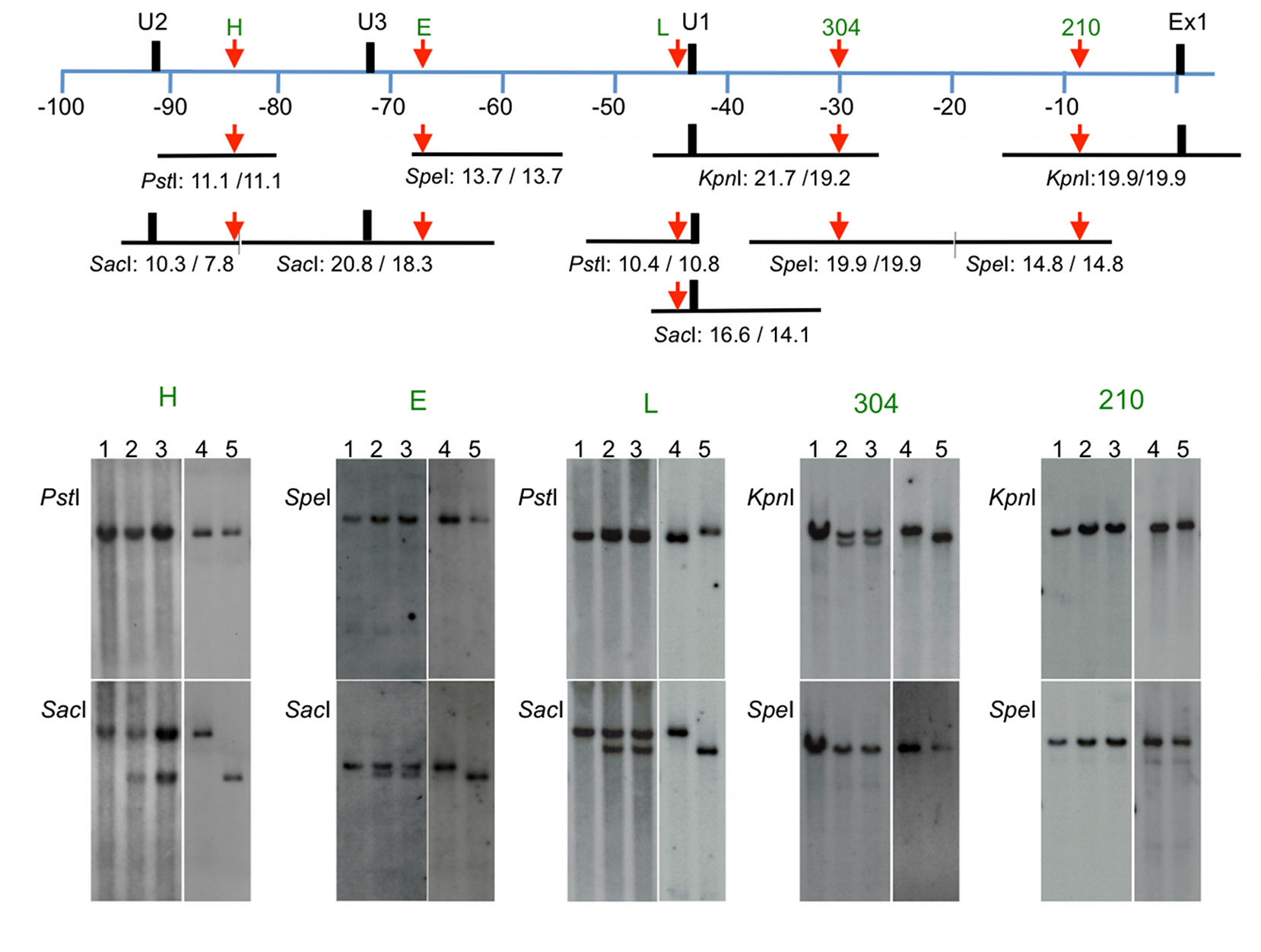

Supplement: Figure S4 — The single copy 425D18 derived BAC transgenes are intact and not rearranged. The topmost part of the figure shows 100 kb upstream of Snrpn exon 1. Black rectangles indicate the three U exons present in the 425D18 BAC as well as Snrpn exon 1. The locations of five probes, termed H, E, L, 304, and 210, are shown in red (arrows). Horizontal lines indicate the lengths and locations of expected fragments for each restriction endonuclease and probe combination. Below each line is the expected size of that fragment, first from the BAC containing U exons, 425Δ5-7, followed by the BAC from which the three U exons have been deleted, 425ΔU1-U3. Restriction endonuclease digests of either transgenic genomic DNA (lanes 1–3) or purified BAC DNA (lanes 4 & 5) were analyzed by Southern blot. Bands differing from the endogenous alleles are detectable only for fragments that include recombineered deletions of the U exons, indicating the absence of rearrangements within that region. Fragments overlap the entire region with the exception of a 1.4 kb span between the SpeI fragment recognized by probe E and the PstI fragment recognized by probe L. A non-repetitive probe for this short region could not be identified. Lane 1: 425Δ5-7A genomic DNA, Lane 2: 425ΔU1-U3D genomic DNA, Lane 3: 425ΔU1-U3H genomic DNA, Lane 4: 425Δ5-7 BAC DNA, Lane 5: 425ΔU1-U3 BAC DNA. (TIFF) [file pgen.1002422.s005.tiff]
